# Wider Letter-Spacing Facilitates Word Processing but Impairs Reading Rates of Fast Readers

**DOI:** 10.3389/fpsyg.2020.00444

**Published:** 2020-03-17

**Authors:** Sebastian P. Korinth, Kerstin Gerstenberger, Christian J. Fiebach

**Affiliations:** ^1^Department of Psychology, Goethe University Frankfurt, Frankfurt am Main, Germany; ^2^Center for Research on Individual Development and Adaptive Education of Children at Risk (IDeA), Frankfurt am Main, Germany; ^3^Brain Imaging Center, Goethe University Frankfurt, Frankfurt am Main, Germany

**Keywords:** crowding, letter spacing, silent reading, visual processing, eye-tracking

## Abstract

Previous reports of improved oral reading performance for dyslexic children but not for regular readers when between-letter spacing was enlarged led to the proposal of a dyslexia-specific deficit in visual crowding. However, it is in this context also critical to understand how letter spacing affects visual word recognition and reading in unimpaired readers. Adopting an individual differences approach, the present study, accordingly, examined whether wider letter spacing improves reading performance also for non-impaired adults during silent reading and whether there is an association between letter spacing and crowding sensitivity. We report eye movement data of 24 German students who silently read texts presented either with normal or wider letter spacing. Foveal and parafoveal crowding sensitivity were estimated using two independent tests. Wider spacing reduced first fixation durations, gaze durations, and total fixation time for all participants, with slower readers showing stronger effects. However, wider letter spacing also reduced skipping probabilities and elicited more fixations, especially for faster readers. In terms of words read per minute, wider letter spacing did not provide a benefit, and faster readers in particular were slowed down. Neither foveal nor parafoveal crowding sensitivity correlated with the observed letter-spacing effects. In conclusion, wide letter spacing reduces single word processing time in typically developed readers during silent reading, but affects reading rates negatively since more words must be fixated. We tentatively propose that wider letter spacing reinforces serial letter processing in slower readers, but disrupts parallel processing of letter chunks in faster readers. These effects of letter spacing do not seem to be mediated by individual differences in crowding sensitivity.

## Introduction

Recent reports that increased spacing between letters improved reading accuracy and oral reading speed of readers with dyslexia instantly, that is, without training (Perea et al., 2012; Zorzi et al., 2012; see also Spinelli et al., 2002), attracted a lot of attention. Several authors interpreted this spacing effect as reflecting an unusual sensitivity of dyslexic readers to crowding (Zorzi et al., 2012; Bellocchi et al., 2019; Bertoni et al., 2019), a phenomenon describing visual discrimination difficulties caused by closely spaced contours (Levi, 2008). Importantly, Zorzi et al. (2012) claimed that this increased vulnerability to crowding is specific to dyslexia, since wider letter spacing did not produce significant improvements in a non-impaired control group. This conclusion, however, has been criticized by Skottun and Skoyles (2012) who pointed out that Zorzi et al. (2012) misinterpreted the lack of a significant result in the non-impaired control group as prove for the absence of an effect. And indeed, facilitated word recognition under conditions of increased inter-letter spacing has been reported also for non-impaired readers in experiments using the lexical decision task (Perea and Gomez, 2012a; Perea et al., 2011, 2012; Dotan and Katzir, 2018). In addition, investigations of sentence and text reading using eye movement recordings revealed significantly shorter fixation durations but higher fixation counts (i.e., average numbers of fixations per word) if letter spacing was wider (Perea and Gomez, 2012b; Slattery and Rayner, 2013; Perea et al., 2016; Weiss et al., 2016; Li et al., 2019). In summary, there is by now a growing body of evidence that wider inter-letter spacing affects reading performance and word recognition not only in dyslexic readers but also in non-impaired readers. For a more comprehensive description of additional studies, which also includes an extensive investigation of text-specific parameters (such as font characteristics) that mediate letter-spacing effects, we refer the reader to Slattery et al. (2016).

If crowding sensitivity is indeed the mechanism underlying the effects of letter spacing on reading performance in dyslexia, it is a plausible hypothesis that individual differences in crowding sensitivity should drive the letter-spacing effect also in unimpaired, that is, in typical readers. More specifically, under this assumption, wider letter spacing should be particularly beneficial for slow readers who should also perform poorly in experimental tasks testing for crowding sensitivity. This relationship has, to our knowledge, so far not been investigated directly.

Crowding describes the phenomenon that objects or letters presented in close proximity to each other are perceived as an “unidentifiable jumble” (Pelli, 2008, p. 445). A mathematical formula that describes the relationship of two major determinants of the crowding effect, that is, (a) the distance of flanking objects to a target object and (b) the eccentricity at which the target object appears from foveal vision, is referred to as Bouma’s law (Bouma, 1970). According to this law, crowding is weaker the closer an object is to foveal vision, which, in turn, implies that foveal vision is per definition uncrowded (except for vision impairments such as amblyopia). Crowding in foveal vision is very difficult to detect, since it typically occurs over very small distances between 4 and 6 arc min (Levi, 2008). Nevertheless, the term foveal crowding is widely used despite the difficulty of dissociating it from other phenomena like lateral masking (Levi, 2008; Song et al., 2014).

In light of these considerations, it seems crucial for understanding the source of benefits when letter spacing is manipulated in text reading tasks, to differentiate effects occurring during foveal as opposed to parafoveal vision. The observation of faster lexical decision times for isolated stimuli presented with increased letter-spacing in central vision indicates a locus of facilitating effects in foveal vision (Perea et al., 2011; Perea and Gomez, 2012a). Nevertheless, this does not exclude the possibility that additional benefit from wider letter spacing may also come from enhanced parafoveal vision of adjacent words during sentence or text reading, because larger uncrowded acuity has been associated with faster reading (Frömer et al., 2015). The present study seeks to disentangle the contributions of foveal and parafoveal vision to letter spacing effects, by including independent measures quantifying foveal and parafoveal crowding sensitivity.

Despite the current study’s focus on beneficial effects of letter spacing, it is important to note that wider character spacing was also found to cause disadvantages. A recent eye-tracking study by Bellocchi et al. (2019) reported that an increase in character spacing impaired saccade programming. Specifically, reading impaired as well as typical readers exhibited a rightward shift of their initial landing position away from an optimal position toward the beginning of the word, suggesting a locus of spacing disadvantages in peripheral or parafoveal vision. More likely within the range of foveal vision were reports about slower reaction times in lexical decision tasks due to wider spacing (Vinckier et al., 2006, 2011; Cohen et al., 2008). In the framework of the so-called bigram coding hypothesis, these authors interpret slower reaction times as the disruption of a visual word recognition mode in which letters can be processed in parallel. Consequently, wider character spacing – exceeding a critical threshold of two space insertions between letters – seems to enforce a slow, serial letter processing mode, because arrays of letters (i.e., bigrams, trigrams, or even whole words) are not perceived as cohesive visual objects (Vinckier et al., 2006, 2011; Cohen et al., 2008).

In summary, generally beneficial effects of wider letter spacing were reported for dyslexic and for non-impaired readers, in experiments that required single word recognition as well as passage reading. Although it is widely assumed that these effects are mediated through a reduction of visual crowding, it is still unclear whether individual levels of foveal or parafoveal crowding sensitivity actually cause the observed reading performance boost in an ecologically valid reading situation (i.e., silent text reading). To fill this gap, the current study estimated individual levels of foveal as well as parafoveal crowding sensitivity for a sample of non-reading-impaired young adults and recorded eye movement data during text reading in two spacing conditions (normal vs. wider).

## Materials and Methods

For the purpose of result validation, data and R-scripts used for analyses and for figure generation presented in the following are available at the OSF data repository: https://osf.io/vgze6/?view_only=e4fab3e77ba54d8d8e45b5551a5a449f.

### Participants

Data were initially collected from 30 participants who were students from varying disciplines recruited through advertisements on campus. All participants were native speakers of German, did not have a history of reading impairments or strabismus (amblyopia), had normal or corrected to normal vision, and received monetary compensation of 10 € per hour for their participation. Six participants had to be excluded due to low quality eye movement data (i.e., primarily tracker loss in the lower right quadrant of the visual field)^1^, leaving a final sample of 24 participants (mean age = 21.29; *SD* = 1.60; 19 female) for data analyses. The experimental procedure was approved by the Ethics Committee of the Department of Psychology at Goethe University Frankfurt (# 2014-80K).

### Text Items Reading Experiment

Fourteen texts were randomly chosen from a stimulus set used in a previous study (Korinth and Fiebach, 2018). These texts – newspaper articles covering various topics such as history, social problems, science – were edited to fit on a computer screen as a whole, resulting in text lengths of 155 to 189 words (average = 170.2). For each text item, three multiple-choice questions assessed reading comprehension depth at three levels of increasing complexity (i.e., gist, conceptual associations, and details). Gist refers to a global level of reading comprehension [e.g., What is the text about? (a) surfing at extremely high waves, (b) surfing equipment for professionals, (c) a dramatic surfing accident], whereas conceptual associations demanded deeper text processing that could not rely on picking up keywords [e.g., Why isn’t the surfer entering the waters? (a) waves are too high, (b) waves are not high enough]. Finally, recall of insignificant detail information [e.g., How old is the surfer? (a) 32, (b) 33, or (c) 34] represented the most difficult level.

In an effort to approximate the experimental conditions described in the study of Zorzi et al. (2012), screenshots of the texts were made, making them appear as printed in black on a white sheet of A4 paper, which due to the height of the presentation monitor was cropped at the lower part of the page. Although uncommon for eye-tracking experiments, we used the proportional font Times New Roman (as opposed to the more frequently used mono-spaced fonts like Courier New) at a 12-point print size, also following the procedures described by Zorzi et al. (2012). Each text was presented either at normal inter-letter spacing or, in the wide text condition, at an inter-letter spacing increased by 2.5 points. Due to the proportional font, unitary values for letter width and center to center distance between letters were not available. Instead, each individual letter width was measured as the number of pixels between its leftmost and rightmost edge; midpoints of width values served as the letters’ center and provided the basis for center to center values between consecutive letters. Converted into units of visual angle, letters were on average 0.24° (*SD* = 0.07) wide. Mean center to center distances were 0.26° (*SD* = 0.05) and 0.38° (*SD* = 0.05) in the normal and the wide spacing condition, respectively. High resolution images of all text items in both spacing conditions as well as the R-scripts used to calculate width and distance measures are available in the OSF data repository accompanying this manuscript.

In contrast to Zorzi et al. (2012) no extra line spacing was applied in the current study, because differences in the difficulty of executing return sweeps to the beginning of a line might have added additional variance. Important in this context is also that texts in the wider letter-spacing condition covered on average 17.25 lines (*SD* = 0.78), whereas normal texts comprised 11.63 (*SD* = 0.50) lines.

### Reading Experiment

Using the tower-mount setup of an EyeLink1000 eye tracker (SR Research, Canada), eye movements were recorded from the right eye at a sampling rate of 1,000 Hz. Participants sat in a height-adjusted chair with their head placed on a chin and forehead rest assuring a constant distance of 50 cm between the screen center and the reader’s eye. Text items were presented using the software Experiment Builder (SR Research, Canada) on a 22-inch (47.5 cm × 30 cm) monitor (LG Flatron E2210; 1680 pixels × 1050 pixels).

Each experiment started with a standard 9-point calibration and validation. If the validation procedure indicated no deviation larger than 1° visual angle in any position, the experimenter started the trial sequence. An initial fixation point appeared at the upper left corner of the screen at the beginning of each trial, indicating the position of the text’s first word. Actual text presentation was initiated by the experimenter through button press when a stable fixation was detected on this fixation point. Participants were instructed to read the text as fast and as accurately as possible and to fixate a second point at the end of the text to indicate that they had completed reading. Subsequently, five multiple-choice questions appeared on the screen in a fixed order. These included the three questions testing comprehension at the gist, conceptual associations, and detail level (see above), which were followed by two ratings (3-point scale: high, medium, and low) of prior knowledge concerning the text’s topic and of how interesting the text was.

Responses were given by pressing the arrow keys on a standard keyboard, that is, left, down, and right for response options a, b, c or high, medium, low, respectively, depending on the question. Participants kept their fingers on the three keys throughout the experiment, which reduced head movements because participants did not have to search for appropriate response keys. There was no time limit for responses to the questions; each answer triggered the appearance of the subsequent question. After the final question was answered, the appearance of the fixation point in the upper left corner indicated the beginning of a new trial. Deviations larger than 1° of visual angle between initial fixation point and gaze triggered the repetition of the calibration and validation procedure.

Each participant read fourteen texts and responded to the corresponding questions. Half of the texts were presented using standard letter spacing, and half appeared with wider letter spacing as specified earlier. Two lists were created in which half of the texts were randomly assigned to the normal and the other half to the wide spacing conditions. Participants saw one of these two lists, and the order of text items was individually randomized for each participant.

### Parafoveal Crowding Sensitivity

**FIGURE 1:**
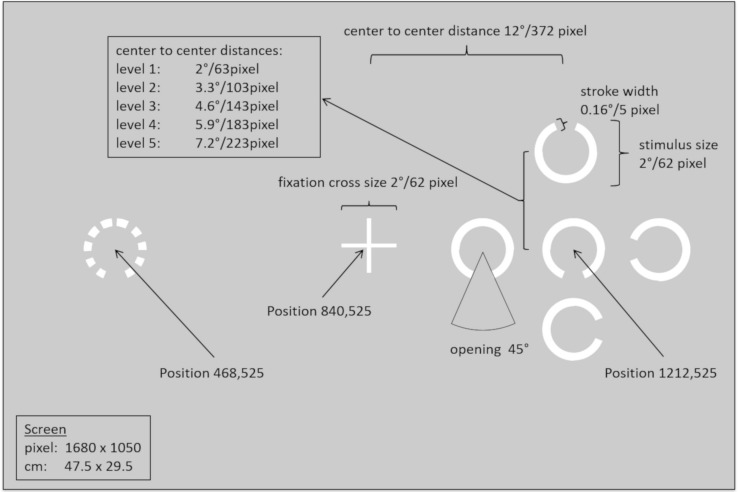
Schematic of stimulus dimensions, distances, and positions for the parafoveal crowding task.

We used a modified version of a task described in Tadin et al. (2012), that estimates individual levels of parafoveal crowding sensitivity for each participant. The task required identifying the direction (i.e., left, right, up, or down) in which a 45°-degree gap of a C-shape (size 2°) pointed. Stimuli were presented in sets of five adjacent C-shapes (white on gray background; RGB 204, 204, 204; see Figure 1) either to the left or to the right of a central fixation cross (center-to-center distance 12° from central fixation position). Responses indicating the gap orientation were only required for the central C-shape (i.e., the target); gap orientations for the four surrounding C-shapes were irrelevant and randomly aligned to one of the four possible orientations.

Crowding levels were experimentally manipulated by varying the distance of the four C-shapes surrounding the target at five levels (i.e., center-to-center distance from the target: 2, 3.3, 4.6, 5.9, or 7.2°). Those five levels were chosen to cover the range from the smallest possible distance (one pixel) between C-shapes at level 1 to an uncrowded spacing condition according to Bouma’s law (Bouma, 1970) at 7.2° (given the fixed eccentricity of 12°), and linearly increasing levels of spacing between these two endpoints. Participants completed 200 trials in total, with half of the stimuli presented to the left and right visual field, respectively. For each combination of hemifield and crowding level, 20 trials were presented. These were distributed equally across all four possible gap orientations.

To ensure that participants kept their gaze on the central fixation cross, gaze position was monitored using the eye-tracker (see above for details on equipment, presentation software, and calibration routine). Each trial started with the presentation of the central fixation cross for 100 ms. Only if the eye-tracker detected a fixation in a 100 pixel × 100 pixel area around the central fixation cross, a stimulus appeared either to the left or to the right of the fixation cross for 150 ms. This short presentation duration ensured that participants could not move their eyes toward the stimulus before it disappeared, because the saccade latency, that is, the time required to initiate a saccade is commonly considered to be at least 150 ms long (Rayner, 1998). Subsequently, a question appeared requesting to indicate the target’s gap orientation. Participants responded using the four arrow keys on a standard keyboard. Detailed information on stimulus sizes and distances is provided in Figure 1.

### Foveal Crowding Test

Foveal crowding sensitivity was assessed using low distance Landolt ring eye charts (OCULUS Nahleseprobe; OCULUS Optikgeräte GmbH, Germany) developed by Haase and Hohmann (1982). From a distance of 40 cm – measured using a tapeline – participants had to name the gap orientation of Landolt rings the experimenter pointed at with a wooden stick. Starting at a stimulus size corresponding to a decimal visual acuity of 0.1, test difficulty gradually increased as long as participants could recognize more than 50% of the set size for the given visual acuity level (e.g., at least four out of six Landolt rings in a line with six items). Visual acuity was logged for the highest level at which the participant recognized the majority of gap orientations correctly. The test was conducted for each eye separately in two conditions, that is, with isolated optotypes and under crowding conditions using row optotypes. Since the isolated optotypes condition represents in fact a classical visual acuity test, the difference between visual acuity in the isolated compared to the crowded condition was interpreted as foveal crowding sensitivity.

### Data Analysis

Six eye movement parameters, associated with different stages of word and text processing, served as the dependent variables in the following analyses. Whether a word is fixated or not during first-pass reading is described by the parameter skipping likelihood, which depends primarily on low level factors such as word length, but also on how predictable a word is within a sentence context (Rayner et al., 2011). First fixation durations are associated with early pre-lexical processing, while gaze durations (i.e., the sum of all fixation durations during first pass reading) reflect higher processing steps such as lexical access (Radach and Kennedy, 2013). Total fixation time (i.e., the sum of all fixation durations for all passes) and fixation count are measures associated with semantic integration of words into the context of a sentence or text (Radach and Kennedy, 2013). Similarly, regression likelihood reflects post-lexical integration effort, for instance due to decay of information stored in working memory or due to incongruences in text comprehension (Reichle et al., 2009).

Eye movement data were analyzed using the software DataViewer (SR- Research, Canada). The program’s default algorithms were applied for fixation detection and the definition of areas of interest (AoI) around each word of the 14 text passages. In preparation for an AoI based analysis, fixation data were exported from a total of 57,320 AoIs, i.e., 2,385 AoIs (from 14 texts with 155 to 189 words), for each of the 24 participants. The discrepancy between the expected number of AoIs (i.e., 2,385 × 24 = 52,740) and the exported number (i.e., 57,320) occurred due to line breaks separating hyphenated words into two AoIs in one but not in the other presentation condition, which created 80 additional AoIs for word fragments. Five words and their corresponding AoIs were excluded from analyses in both conditions (minus 200 AoIs). Following a standard procedure, the AoI around the first word of each text item was excluded, so that data points from a further 336 AoIs (14 items by 24 participants) were deleted from the dataset. Also, the first trials for two participants were excluded, because participants made a saccade to the lower right corner immediately after text presentation, thus terminating the trial prematurely (minus 337). Tracker loss in three trials of one participant led to additional exclusions of AoIs (minus 520). Furthermore, on a participant-specific level, AoIs were excluded if they were affected by a blink (minus 4,550), if fixation durations were shorter than 50 ms (minus 140), or longer than 800 ms (minus 21). Final analyses were conducted using 51,216 data points from 2,366 unique AoIs.

R (R Core Team, 2018) and the package lme4 (Bates et al., 2015) were used for calculating linear mixed-effect models (LMM) and generalized linear mixed-effect models (GLMM) for eye movement parameters and for the parafoveal crowding task. The chief advantage of this method is that differences between participants or items are not treated as unexplained variance as is the case in classic *t*-tests or ANOVAs; rather, these variance components are explicitly modeled. Test statistics (i.e., *t-* or *z*-values) of fixed effects (e.g., normal versus wider spacing) allow a straightforward estimation of significance. The package lme4 does not provide *p*-values for *t*-statistics, since there is no consensus on how to calculate denominator degrees of freedom in mixed designs (Bates, 2006). Therefore, statistical significance was determined here using a conventional threshold of absolute *t* > 2 (Kliegl et al., 2010). This does not apply for GLMMs since the model outputs *z* instead of *t*-values.

Significance of random effects, for instance, whether a participant made generally longer fixations (i.e., participant-specific intercept), or the individual strength and direction with which a participant responded to the spacing manipulation (i.e., participant-specific slopes), or the correlation of both, are estimated by comparing models of increasing complexity by means of likelihood ratio tests (Baayen et al., 2008; Kliegl et al., 2010). The rationale of this procedure is that, if the inclusion of a random effect improves the goodness of model fit compared to a model without this random effect (for detailed information see Appendix 1) its inclusion is justified because it leads to a growth in explained variance. Due to the focus of our study on individual differences, we included random intercepts and slopes for participants and single words in addition to the fixed effect factor spacing (normal vs. wide).

## Results

### Reading Experiment – Comprehension and Text Ratings

The low number of data points (i.e., 24 participants × 14 text items in two spacing conditions) available for comprehension measures and text rating impedes the applicability of LMMs, which is why Student’s *t*-tests were applied to test for behavioral differences between the two letter-spacing conditions. Although gist recall was – numerically – slightly better under normal letter spacing conditions (89.29% correct, *SD* = 14.13) compared to wider letter spacing (84.52% correct, *SD* = 12.58), the difference was not statistically significant, *t*(23) = 1.19. Also, performance on the conceptual association questions did not differ significantly between normal (89.19% correct, *SD* = 14.16) and wider letter spacing (90.48% correct, *SD* = 12.40), *t*(23) = −0.35. And finally, recall of small details was not affected by the letter spacing manipulation, namely, 67.36% (*SD* = 12.43) of all questions were answered correctly in the normal spacing condition compared to 69.05% (*SD* = 19.68) with wider letter spacing, *t*(23) = −0.36.

The questions intended to control for possible effects of content bias showed a small but significant advantage for prior knowledge ratings in the normal spacing condition (*M* = 2.59, *SD* = 0.28) in comparison to the wider spacing condition (*M* = 2.41, *SD* = 0.30), *t*(23) = 2.72, *p* = 0.01. No significant difference was found between participants’ ratings of how interesting they found texts in the normal spacing condition (*M* = 2.16, *SD* = 0.36) compared to the wider condition (*M* = 2.10, *SD* = 0.30), *t*(23) = 0.88.

### Reading Experiment – Fixed Effect Estimates

Using the three duration measures, that is, first fixation duration, gaze duration, and total fixation time as dependent variables, LMMs were computed for which the independent variable letter-spacing (normal vs. wide) was included as the fixed effect, and participants and AoIs (i.e., single words indexed by text ID and word position) were modeled as having random intercepts and slopes. All three models (i.e., one per dependent variable) were computed twice, once using the log-transform of the duration measures (to take care of possible violations of preconditions for computing LMMs, given that fixation durations follow a right skewed distribution) and once using the un-transformed measures. The latter allows for a more intuitive interpretation of the results.

**FIGURE 2:**
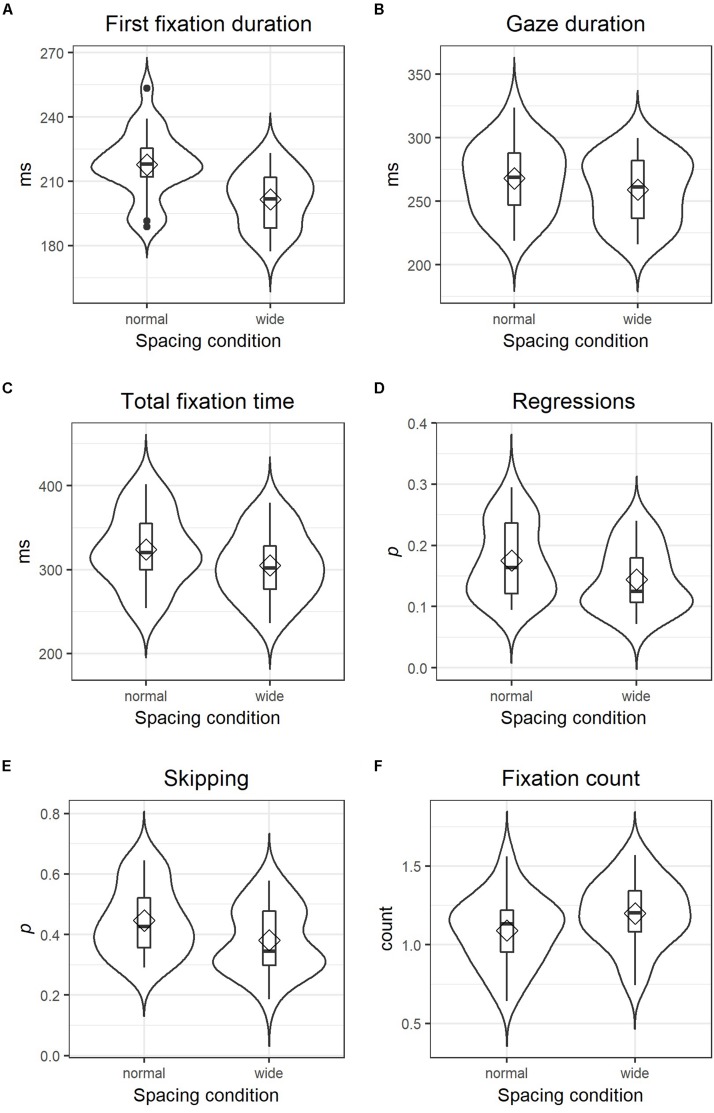
Combined violin and box plots of three duration measures **(A–C)**, two probability measures **(D,E)** and one count measure **(F)** in both spacing conditions; diamonds indicate mean values and horizontal bars indicate the median. The filled circles in **(A)** represent outliers below the first and above the third quartile. Note that values presented here are not (G)LMM estimates but simple mean calculated from values averaged separately for each participant in each spacing condition. Probability values as for instance in **(D)** are calculated by averaging over all AoIs (for each participant and condition separately) whether it received a regression or not corresponding to 1 and 0, respectively.

First fixation durations were significantly reduced in the wider letter spacing condition using both log-transformed (*t* = −10.05) or un-transformed values (*t* = −10.97) as the dependent variable, see Table 1. The un-transformed measure provides the information that the model estimates the intercept (i.e., first fixation duration under normal spacing conditions) at 217.31 ms and the effect of wider spacing as a reduction of fixation durations by 15.81 ms. Model estimates presented here and in Table 1 are in good agreement – albeit not identical – with mean values (aggregated across words and items by participant and spacing condition) plotted in Figure 2A.

**TABLE 1:** Fixed effect results of linear mixed-effect models for eye movement duration measures.

			Beta	*SE*	*t*	*Sig.*
Log-transformed	First fixation duration	Intercept	5.31	0.01	370.73	*
		Condition wide	−0.07	0.01	−10.05	*
	Gaze duration	Intercept	5.44	0.02	285.49	*
		Condition wide	−0.03	0.01	−4.09	*
	Total fixation time	Intercept	5.59	0.03	219.64	*
		Condition wide	−0.05	0.01	−6.99	*
Untransformed	First fixation duration	Intercept	217.31	3.17	68.57	*
		Condition wide	−15.81	1.44	−10.97	*
	Gaze duration	Intercept	260.19	6.28	41.42	*
		Condition wide	−7.20	1.89	−3.80	*
	Total fixation time	Intercept	311.67	9.87	31.58	*
		Condition wide	−14.97	2.34	−6.40	*

**The R package lme4 does not provide p-values; here, absolute t-values larger than 2 are assumed to indicate statistical significance, which is marked by an asterisk in the table. As outlined in more detail in the section “Materials and Methods,” we report LMMs for both, that is, log-transformed data to account for non-normal distribution and untransformed data to allow for a direct interpretation of parameter estimates*.*

Gaze durations were also significantly shorter in the wider spacing condition (−7.20 ms) compared to normal spacing (260.19 ms), *t* = −3.80 for untransformed, and *t* = −4.09 for log-transformed values, and total fixation times were reduced by 14.97 ms during reading of texts with wider letter spacing in comparison to normal spacing (311.67 ms), *t* = −6.40 untransformed and *t* = −6.99 for log-transformed values (see also Figures 2B,C and Table 1 for detailed statistics).

For the likelihood of regressions and the likelihood of skipping, generalized linear mixed-effect models (GLMMs) were calculated, which assume a binomial function (i.e., a word was skipped = 1, or not = 0). The independent variable structure was identical to the LMMs calculated for duration measures, that is, spacing (normal vs. wide) represented the fixed effect, whereas participants and AoIs were modeled as having random intercepts and slopes. A direct interpretation of coefficients from GLMMs (see Table 2) is difficult, since model outcomes are given as logit values (i.e., the logarithm of odds). The recalculation to odds through exponentiation and the conversion to probabilities [*p* = odds/(1 + odds)] provide more intuitively comprehensible values: the intercept coefficient for regressions of −1.75 corresponds to an odds value of 0.17 and a probability of 0.15. In other words, the probability that participants made a regression to a word was 15% in the normal spacing condition. Adding the estimate for the effect of wider spacing (i.e., −0.27) to the intercept estimate (i.e., −1.75) provides a logit estimate of −2.02 for the occurrence of a regression in the wider letter spacing condition, which corresponds to a probability of 0.12. This decrease of regression likelihood due to wider letter spacing is nicely illustrated in Figure 2D and was found to be statistically significant, *z* = −5.40, *p* < 0.001. Note, that the plots in Figure 2 are based on mean values aggregated across words and items by participant and spacing condition, which are not necessarily identical to model estimates. Skipping rates also dropped significantly in the wider letter spacing condition, *z* = −5.68, *p* < 0.001. Applying the conversion rules of logit values to probabilities, we can interpret the estimates of −0.29 (intercept) and −0.33 (spacing effect) as skipping probabilities of 0.43 under normal spacing conditions and 0.35 in the wider spacing condition (see also Figure 2E for mean values aggregated across words and items by participant and spacing condition).

**TABLE 2:** Fixed effect results of generalized linear mixed-effect models for regressions, skipping, and fixation count.

		Beta	*SE*	*z*	*p*
Regressions	Intercept	−1.75	0.09	−19.51	<0.001
	Condition wide	−0.27	0.05	−5.40	<0.001
Skipping	Intercept	−0.29	0.11	−2.54	0.011
	Condition wide	−0.33	0.06	−5.68	<0.001
Fixation count	Intercept	−0.03	0.04	−0.67	0.503
	Condition wide	0.10	0.01	9.02	<0.001

**A direct interpretation of coefficients from GLMMs is difficult, since model outcomes represent the logarithmic transform of odds, and incidence rates for the probability measures Regression and Skipping, and the count measure fixation count, respectively. We have recalculated the beta coefficients into probabilities, as described in detail in the text.**

Lastly, the GLMM computed for fixation count assumed a Poisson distribution. In contrast to all previous models, the most complex random effect definition returned an over-fitted model, which is why AoIs were in this analysis just modeled as having random intercepts and no random slopes. Similar to likelihood measures, coefficients for the GLMMs computed for count measures presented in Table 2 are difficult to interpret directly, as they are the logarithmic transforms of incidence rates. However, the exponentiation of the intercept estimate (i.e., −0.03) results in a more intuitively comprehensible incidence rate value of 0.97 in the normal spacing condition. Adding the estimated effect of wider character spacing (i.e., 0.10) to the intercept and its exponentiation leads to an estimated incidence rate of 1.07 for fixation counts in the wider spacing condition. In short, wider letter-spacing led to significantly higher fixation counts (*z* = 9.02, *p* < 0.001; see also Figure 2F and Table 2).

### Reading Experiment – Random Effect Estimates

(G)LMMs for the analysis of eye movement parameters included random effects terms modeling random intercepts and random slopes for each participant and word. The inclusion of each random intercept, slope, and their correlations was justified because – with the exception of fixation counts – it led to significant improvements of model fit (see section “Materials and Methods, Data Analysis” for more details). Appendix 1 provides comprehensive information about the goodness of model fit for all eye movement measures at different levels of model complexity.

The correlation of intercepts and slopes is informative, since it provides insights about which words (AoIs) and participants were more strongly or weakly affected by the letter spacing manipulation. Table 3 summarizes all random effects correlations. No correlation of word-specific intercepts and slopes can be provided for fixation count (last row of Table 3) due to overfitting problems when this correlation term was included into the model.

**TABLE 3:** Correlations of random effect estimates for word- and participant-specific intercepts and the corresponding slopes.

	Words	Participants
First fixation duration	−0.65	−0.45
Gaze duration	−0.17	−0.29
Total fixation time	−0.09	−0.48
Regressions	−0.52	−0.25
Skipping	−0.32	−0.25
Fixation count	−	−0.78

**No correlation for word-specific intercepts and slopes can be reported for the analysis of fixation counts, because the model that included this correlation term indicated an overfitting. Also, no p-values indicating significance of the here presented correlations are provided; instead, the inclusion of the correlation term is justified by a significant increase of model fit compared to a model without this term (see Appendix 1 for details).**

Most relevant for the current study is the finding that all participant-specific intercepts of duration measures (i.e., first fixation duration, gaze duration, and total fixation time) correlated negatively with their slope estimates. This indicates that participants with generally longer fixation durations (i.e., with a higher intercept) exhibited a stronger decrease of fixation durations (i.e., a steeper slope) if letter spacing was wider.

For the likelihood measure of regressions, participant-specific intercepts and slope correlations indicate that participants who are generally prone to regressions showed a reduced likelihood of regressions if letter spacing was wider. Similarly, participants who generally skipped words more often, exhibited less skipping under wider letter spacing conditions. The highest correlation was found for fixation count, indicating that the fewer fixations a participant makes in general, the more her or his fixation count was increased by wider letter spacing.

### Reading Experiment – Word Length Effects

**FIGURE 3:**
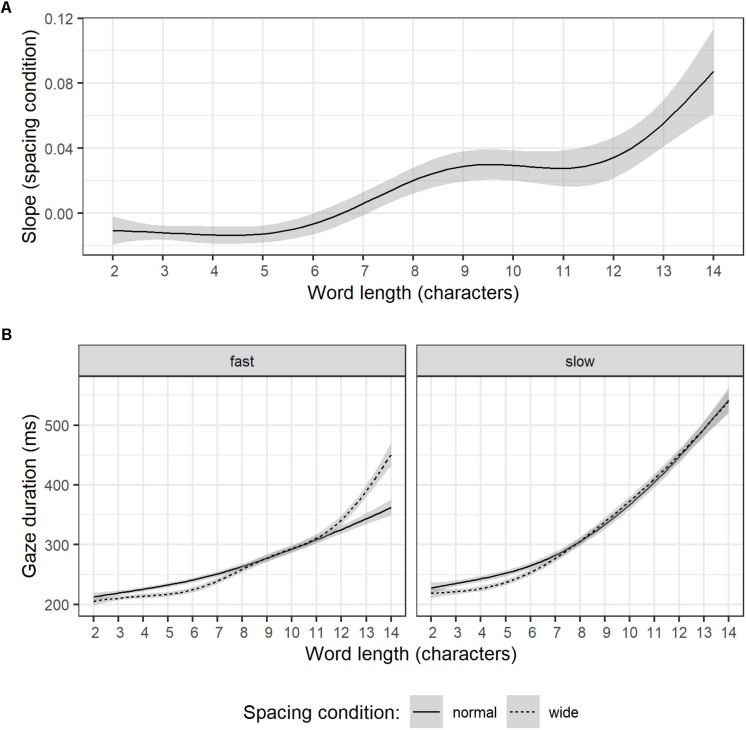
Word specific slopes estimates of character spacing (LMMs for gaze duration) as a function of word length **(A)**. Illustration of word length effects on gaze duration for relatively fast (left) and slow readers (right) for normal (solid line) and wide (dashed line) character spacing **(B)**. Note, words longer than 14 characters were omitted because too few items per length category were available. Error corridors represent the pointwise 95% confidence interval on the fitted values of the generalized additive model smooth lines.

Following up on a reviewer comment, additional analyses were carried out *post hoc*. In controlled single word recognition experiments, word length was found to mediate spacing effects (Vinckier et al., 2011). Although gaps between letters were considerably larger in these experiments (i.e., two space insertions, which, according to the authors, may represent a critical threshold for abolishing parallel processing of adjacent letter strings) than the manipulation applied in the current study, an investigation of spacing effects for different word lengths might be informative. Given that gaze duration is a widely accepted indicator of lexical processing effort (Radach and Kennedy, 2013), we reasoned that this eye-tracking parameter is most likely to be affected by word length. As described earlier (see section “Reading Experiment –Random Effect Estimates”), LMMs presented in the current study included random intercepts and slopes for each word, providing estimates of how wider spacing affected gaze durations in every word (i.e., word-specific slopes), which can consequently be correlated with word length. A small to moderate correlation between word length and word-specific slope estimates was evident, *r* = 0.25, *p* < 0.001. Furthermore, the absence of a trend between words of lengths 2 to 5 characters visible in Figure 3A suggests that this relation is not necessarily linear. Gaze duration for words of less than seven characters had negative slope estimates (i.e., wider spacing reduced gaze durations), whereas for words of more than seven characters, gaze durations were longer in the wider spacing condition.

The interaction of word length and spacing effects is particularly interesting from an individual differences perspective. Namely, reading proficiency for readers of the shallow German orthography has been linked to the magnitude of how word length affects eye movement parameters in studies contrasting typical with reading impaired participants (Hawelka et al., 2010; Gagl et al., 2015) or beginning with proficient readers (Rau et al., 2014; Tiffin-Richards and Schroeder, 2015). In the context of the dual-route model of visual word processing (Coltheart et al., 2001) the typically reported stronger impact of word length for less proficient readers is attributed to more pronounced reliance on slow, serial grapheme to phoneme processing for words that proficient readers can approach through a direct orthographic route.

Attempts to further unravel the interaction of word length and spacing effects through including participant-specific random intercepts and slopes for the interaction of word length × spacing condition into an LMM failed, due to convergence problems. Alternatively, a simpler version that modeled log-transformed gaze duration as depending on the fixed effect terms word length, spacing condition, and their interaction as well as on a random effect term which assumed participant-specific random intercepts and random slopes for word length, converged. Results indicate significant effects of word length (beta = 0.14, *SE* = 0.01, *t* = 14.98), spacing condition (beta = −0.03, *SE* = 0.01, *t* = −7.17), and the interaction of word length × spacing condition (beta = 0.03, *SE* = 0.004, *t* = 7.09). In addition, a positive correlation of 0.37 for participant-specific random intercepts and slopes indicates that readers with generally longer gaze durations were especially slowed down through increasing word length. The comparison of this model to a model that only accounted for random intercepts for participants indicated significantly better model fit indices (χ^2^ = 296.52, *df* = 2, *p* < 0.001), thus, justifying the inclusion of the random effects correlation.

Supplemental, purely descriptive data exploration is provided in Figure 3B. Participants were classified as relatively fast and slow readers by a median split of reading rate under normal character spacing conditions (words per minute). For each reader group gaze duration is plotted depending on word length during reading under normal spacing conditions (solid line) and wider character spacing (dashed line). In line with the random effect correlation in our LMM that accounted for word length effects, gaze durations of relatively slow readers were apparently more affected by word length than gaze durations of relatively fast readers. On words shorter than 7 letters gaze durations during wider character spacing were shorter for both groups. However, whereas slower readers’ gaze durations on words longer than 7 letters were seemingly unaffected by wider spacing, faster readers exhibited an abrupt gaze duration increase for words longer than 12 letters. Note, however, that only few items were available for words longer than 14 characters, leading to large standard errors of estimates for these words. Consequently, LMM results and Figures presented in this section are based on a data subset omitting words longer than 14 characters.

### Reading Experiment – Words per Minute

**FIGURE 4:**
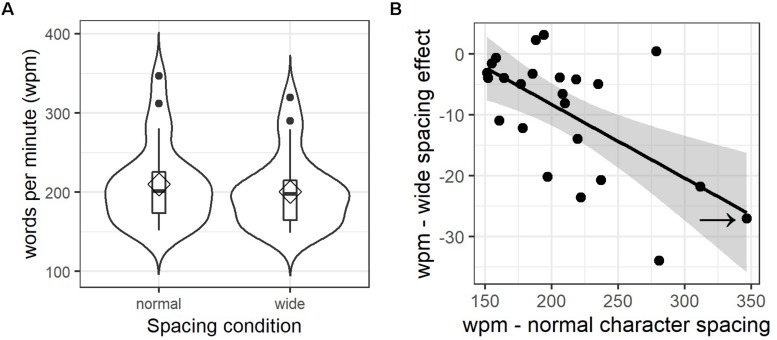
Letter spacing effects on reading rate. **(A)** Combined violin and box plots of words per minute values, aggregated by participant and spacing condition; diamonds indicate mean values, horizontal bars indicate the median. Filled circles in **(A)** represent outliers above the third quartile. **(B)** Scatterplot illustrating the correlation of mean words per minute (wpm) values under normal spacing (*x*-axis) with individual differences in the letter spacing effect (wide minus normal condition; *y*-axis). Error corridors represent the pointwise 95% confidence interval on the fitted values of the linear regression (black line). The arrow points at the example participant described in the text.

While the analysis of each eye movement parameter provides insight into fine-grained mechanisms of reading, it does not represent an ecologically valid and intuitively comprehensible estimate of general changes in reading performance caused by wider letter spacing. Therefore, we also calculated the words read per minute (wpm), defined as the number of words per text divided by the total text reading time, separately for each item, condition, and participant. The direct comparison of both conditions reveals a significant disadvantage for wider letter spacing (*M* = 200.43 wpm, *SD* = 45.93) compared to the normal spacing condition (*M* = 209.88 wpm, *SD* = 51.49), *t*(23) = 4.62, *p* < 0.001. In other words, participants read on average 9.45 words less per minute when letter spacing was wider (see Figure 4A). Nevertheless, whereas most participants’ performance suffered through wider spacing, others did show a small benefit. This effect becomes clear as individual difference values (i.e., average wpm for normal subtracted from wider letter-spacing) are correlated negatively with individual wpm values of the normal spacing condition, *r* = −0.62, *t*(22) = −3.73, *p* < 0.01. Figure 4B illustrates this correlation. For example, a very fast reader (marked with a black arrow in Figure 4B) who read on average 347 words per minute under normal spacing conditions showed a strong decrease in reading rates of 27 words less per minute when letter-spacing was wider. Clearly, it would have been more elegant to run these analyses using linear mixed-effect modeling to take the correlation of random participant intercepts and slopes into account; however, as for comprehension measures and text ratings, the number of available data points is insufficient for LMM calculations. Attempts resulted in a singular fit outcome, which is strongly suggestive of overfitting and led us to not report these results.

### Parafoveal Crowding Task – Fixed Effect Estimates

**FIGURE 5:**
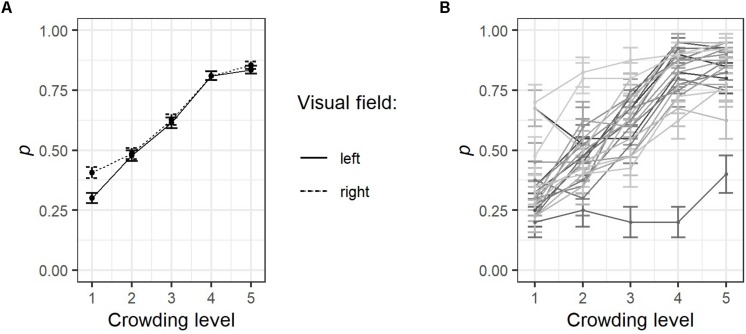
Results of the parafoveal crowding task. **(A)** The probability (p) that a target stimulus was recognized or not was coded as 1 or 0, respectively. Separately for each visual field and crowding level, values were averaged over all participants and trials. **(B)** Line graphs representing single participants’ average response accuracy for each crowding level, error bars indicate ±1 SE. For details on the parafoveal crowding task, see Figure 1. Note that the mean values presented here are not necessarily identical to the GLMM estimates presented in Table 4.

Response accuracy, that is, the likelihood that participants spotted the correct direction of the central circle’s gap, was lower for crowded (level 1) than for uncrowded stimulus presentation conditions (level 5), see Figure 5A. Figure 5B shows line graphs of each participant, which makes the broad variability between participants’ responses to the crowding manipulation clearly visible.

Interestingly, in the highest crowding condition, stimulus presentation to the right visual field resulted in a better response accuracy (*M* = 0.41 *SD* = 0.49) in comparison to stimulus presentation to the left visual field (*M* = 0.30, *SD* = 0.46). A generalized linear mixed-effect model (GLMM) was calculated, which assumed a binomial function of the dependent variable (i.e., a response was either correct = 1 or incorrect = 0). The independent variable crowding level was modeled assuming a linear relationship interacting with the fixed effect visual field (left vs. right). Participants were assumed to have random intercepts and random slopes for crowding level.

Results presented in Table 4 confirm the pattern visible in Figure 5A, that is, response accuracy improved with increased spacing between circles, *z* = 12.14, *p* < 0.001. Also, as indicated by the significant effect for presentation side, *z* = 2.56, *p* = 0.010, presentation to the right visual field created an advantage (i.e., higher response accuracy). The latter finding might be a data distortion problem: Although participants were instructed to fixate a cross at the screen center (pixel coordinates 840, 525), they may have shifted their gaze preferentially to the right, thereby creating a small benefit during trials in which the target appeared to the right of fixation. However, fixation positions prior to stimulus presentation in the level 1 crowding condition were aligned slightly left to the central fixation point with no obvious shifts to any direction if stimuli were presented to the left (*M* = 834.73, *SD* = 30.70) or to the right (*M* = 833.91, *SD* = 32.25), *t*(928) = 0.40, ns.

**TABLE 4:** Fixed effect results of generalized linear mixed-effect models for parafoveal crowding task.

	Beta	*SE*	*z*	*p*
Intercept	−1.59	0.17	−9.42	<0.001
Crowding level	0.73	0.06	12.14	<0.001
Side (right)	0.39	0.15	2.56	0.010
Crowding level × side	−0.09	0.05	−1.72	0.086

**GLMM beta coefficients presented here are logits (i.e., logarithm of odds). These values are easier to understand if converted into odds through exponentiation and recalculated as probabilities (p = odds/1 + odds). Accordingly, the intercept estimate can be read as: the probability that a target stimulus was identified at the highest crowding level was 0.17. For each increase of spacing between target and flanker stimuli the logit value of 0.73 has to be added to the logit intercept estimate, which translates into probability values of 0.30, 0.47, 0.65, and 0.79 at the crowding levels 2, 3,4, and 5, respectively*.*

### Linking Foveal Crowding, Parafoveal Crowding, and Letter Spacing Effects

**FIGURE 6:**
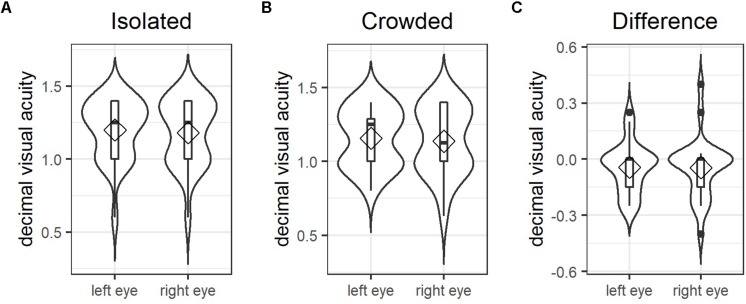
Foveal crowding sensitivity: combined violin and box plots illustrating between participant variance in visual acuity for both eyes measured using **(A)** isolated optotypes and **(B)** crowded optotypes. Panel **(C)** shows individual difference values calculated as the visual acuity for isolated minus crowded optotype identification, which serves as the estimate for individual levels of foveal crowding sensitivity. Diamonds indicate mean values; horizontal bars indicate the median; filled circles in **(C)** represent outliers above the third and below the first quartile.

One of the main aims of this study was to examine whether individual levels of crowding sensitivity account for individual differences in how participants responded to the letter spacing manipulation. As illustrated in Figure 6A, visual acuity for isolated optotypes was age-appropriate for the sample of young adults tested here, that is, well above 1.00 (Colenbrander, 2008). Figure 6B shows that between-participant variance increased considerably when crowded optotypes had to be identified. Difference values (i.e., visual acuity of crowded minus isolated optotypes; see Figure 6C) were computed for each participant and served as estimates for individual foveal crowding sensitivity.

(G)LMMs for eye movement parameters included random intercepts and slopes for each participant, to provide individual estimates of each participant’s general performance (i.e., intercept) and of how strong and in which direction the spacing condition affected her/his eye movements (i.e., slope). These estimates were exported and correlated with individual performance scores in the foveal and parafoveal crowding task. None of the crowding tasks correlated significantly with any of the participant-specific slope estimates for the eye movement parameters (all absolute *r* > −0.27, *p* > 0.21). The only tendency for an effect became apparent for the correlation of foveal crowding sensitivity with regression likelihood, *r* = −0.38, *p* = 0.06, which, however, would be far from surviving correction for multiple testing (e.g., corrected threshold according to Bonferroni method: *p* < 0.002). One participant showed an unusual pattern, performing at chance level for four crowding levels, see Figure 5B. In order to exclude that this outlier distorted the results, all correlations were computed once with (Table 5) and once without this participant (Table A2 in the Appendix 2). The exclusion of this participant did not change the observed pattern of results.

**TABLE 5:** Correlations of visual acuity, foveal and parafoveal crowding with individual levels of spacing effects for six eye movement parameters, *p*-values in parentheses.

	Visual acuity	Foveal crowding	Parafoveal crowding intercept	Parafoveal crowding slope
First fixation duration	0.23 (0.29)	0.17 (0.44)	−0.24 (0.26)	0.19 (0.38)
Gaze duration	0.14 (0.51)	0.07 (0.75)	−0.27 (0.22)	0.27 (0.21)
Total fixation time	0.10 (0.66)	0.04 (0.85)	−0.18 (0.40)	0.08 (0.70)
Regressions	−0.26 (0.22)	−0.38 (0.07)	0.05 (0.82)	0.03 (0.90)
Skipping	−0.27 (0.21)	0.02 (0.94)	−0.03 (0.91)	0.21(0.32)
Fixation count	−0.03 (0.90)	−0.21 (0.32)	0.02 (0.94)	−0.22 (0.30)

## Discussion

Our study shows that for a sample of young, German-speaking adults without a history of reading impairments, printing words in wider letter spacing affected word recognition, reading rate and individual readers heterogeneously. With respect to single word processing as examined using eye tracking, fixations were indeed significantly shorter than in the normal spacing condition. This, however, did not translate into an overall improvement of reading rate, as measured here by counting the words read per minute. On the contrary, we observed a significant reduction in the reading rate, across the sample of 24 participants that was especially pronounced for relatively fast readers. Importantly, text comprehension was not affected by the letter spacing manipulation.

### Overall Reading Performance

Overall reading rates were significantly reduced under conditions of increased letter spacing. One trivial source for this effect is that wider letter spacing covered more text lines and therefore demanded more return sweeps. Also, the shorter duration of fixations quite obviously reflects that less information can enter through foveal vision if letters are expanded over a larger part of the visual field in the wider letter spacing condition. This, as a consequence, also generated the need for additional fixations, leading to the observed effect of slower reading rates despite shorter fixation durations, which replicates previous reports for non-impaired readers of Spanish (Perea and Gomez, 2012b; Perea et al., 2016), Hungarian (Weiss et al., 2016), and English (Slattery et al., 2016; Li et al., 2019).

An important novel result of the current study is that the strength of the letter spacing effect is mediated by inter-individual differences in reading proficiency: whereas faster readers can suffer substantially from wider letter spacing (i.e., up to 20 to 30 words per minute), slower readers show no or comparably weak effects (Figure 4B).

### Eye Movement Parameters

A more detailed picture emerges when the contribution of each eye movement parameter is considered. The strongest reduction of fixation durations through wider letter spacing (−15.81 ms) was observed for first fixation durations, see Table 1. This parameter has been associated with pre-lexical rather than lexical or semantic processing of visually presented words, such as orthographic processing (Radach and Kennedy, 2013). While also significantly affected by letter spacing, the reductions found for gaze duration (−7.2 ms) and total fixation time (−14.97 ms) were smaller. Because these latter two parameters are composite measures that comprise the duration of the first fixation, it seems reasonable to conclude that fixation durations are largely reduced through facilitated low level word processing. A closer look at the LMMs’ random effect results reveals that the participant-specific random intercepts and slopes are correlated for all eye movement parameters (right column, Table 3). For the case of fixation durations, this indicates that especially slower readers – even if not diagnosed with reading impairments – can benefit from increased letter spacing in terms of single word processing times.

The participant specific correlations of intercept and slope were also found in those eye movement parameters that contributed to prolonged reading rates, that is, the fixation count and the probability of word skipping. Since higher skipping likelihoods (e.g., for short and highly predictable words) contribute to faster reading rates, the negative correlation we have observed for these two parameters indicates that participants that show high rates of skipping under conditions of normal word presentation, showed greatest reductions in skipping behavior and were, as a consequence, forced to make more fixations. Increasing the letter spacing, thus, particularly impaired fast and efficient readers.

An elaboration of what exactly triggered the reduction of skipping likelihoods for most participants seems in order. Skipping can occur as the result of parafoveal preview of words to the right of the currently fixated word (Rayner et al., 2011; Schotter et al., 2012). Increasing the spacing between letters has the obvious effect that fewer letters fall into parafoveal vision and that – due to the increase of inter-word spacing – the distance to subsequent words becomes larger. This most likely necessitates fixations on words, which were otherwise adequately recognized in parafoveal vision and therefore under normal spacing conditions be skipped.

In addition, as suggested already by Bellocchi et al. (2019), saccade planning is likely disturbed due to the wider spacing. Coarse parafoveal information about physical characteristics (e.g., word length) of upcoming words provides valuable information about the optimal landing position on a word (Brysbaert et al., 1996) but may not be available to the same degree under wider letter spacing. If suboptimal landing positions were the chief mechanism causing the observed fixation count increase, this would have caused corrective saccades within the same word, which, in turn, should have been reflected in longer gaze durations. However, gaze durations were in fact shorter when letter spacing was wider. This seems to suggest that the observed increase in fixation count is not caused by additional fixations on each word, but rather – as indicated by the decrease in skipping likelihood – by fixations on words that would not be fixated under conditions of normal letter spacing.

This interpretation, however, must be revised if word length effects are taken into account. *Post hoc* analyses correlating word-specific slope estimates of gaze duration with word length indicate that facilitated lexical processing (i.e., shorter gaze duration) due to wider spacing occurred primarily for shorter words (see Figure 3A). This finding is further supported by a significant interaction of the fixed effect terms word length × spacing condition in an LMM that accounted for word length effects. The random effect correlation of the latter model also indicates stronger word length effects for slower readers. And finally, a disruptive impact of wider spacing (i.e., increased gaze durations) on longer words was – albeit merely descriptively – visible only for faster readers in Figure 3B. This result pattern fits well with the core idea of the bigram coding hypothesis (Vinckier et al., 2006, 2011; Cohen et al., 2008) and findings that less proficient readers rely more often on slow serial processing (Hawelka et al., 2010; Rau et al., 2014; Gagl et al., 2015; Tiffin-Richards and Schroeder, 2015). Thus, we propose that wider character spacing affects slower readers less or not, since it facilitates and therefore reinforces a preexisting serial processing preference. In turn, the disruption of parallel visual processing (as proposed by Vinckier et al., 2006, 2011) is stronger for more proficient readers. In conclusion, in addition to more frequent return sweeps and fixations on words that were skipped under standard spacing conditions, disruptive effects of parallel visual word recognition due to wider character spacing are likely another factor explaining the decrease of overall reading rates for faster readers.

### Crowding and the Letter Spacing Effect

The present study was also intended to examine whether individual differences in crowding sensitivity could explain the effect of letter spacing on reading rates and eye-tracking-markers of reading. Despite our ambition to account for the complex nature of the crowding phenomenon with implications on foveal and parafoveal vision, no association of crowding sensitivity with how wider letter spacing affected any of the eye movement parameters was found. Obviously, the lack of significance does not prove the absence of an effect; given that only 24 participants provided useful data, the lack of an association with crowding sensitivity might be a mere problem of low statistical power and small effect sizes. Also, it cannot be fully excluded that the tests selected to assess crowding do not optimally represent the crucial component that drives the letter spacing effect. For the assessment of parafoveal crowding, numerous procedures and outcome variables have been proposed (Levi, 2008); instead of the here-applied manipulation of flanker distance at a fixed eccentricity with individual intercepts and slopes as outcome variables, a manipulation of target eccentricity might have provided more informative measures.

However, given the strong effect of letter spacing on first fixation durations, it seems most plausible that benefits associated with wider letter spacing are mainly driven by foveal processes. A clear-cut decision whether foveal visual processing difficulties of letters arranged in close proximity are caused by crowding or by contrast masking effects is difficult to make (Levi et al., 2002), since crowding occurs – in the healthy adult fovea – at a very small critical spacing of about 0.05 degrees (Pelli et al., 2016). An alternative interpretation of shorter fixation durations due to wider character spacing has been laid out in the discussion concerning word length effects, namely that wider character spacing might facilitate slow serial word recognition by separating single letters, which should boost word recognition especially for less proficient readers that do not apply a fast parallel processing mode as assumed by the bigram coding hypothesis (Vinckier et al., 2006, 2011; Cohen et al., 2008). This explanation would also account for observed disadvantages in word recognition among fast readers – supposedly caused by disrupted parallel processing of long words. Importantly, this interpretation does not necessarily require assumptions regarding individual differences in the visual domain. Namely, contrast masking effects due to letters in foveal vision might occur for all readers to the same extent. However, they will likely affect readers applying a word recognition mode based on serial (single) letter recognition stronger than those who can rely on parallel recognition of letter arrays. This interpretation challenges the currently predominant hypothesis that individual differences in how letter spacing affects reading behavior depend on individual differences in visual crowding sensitivity. However, we do not want to propose this as unitary mechanism underlying letter spacing effects. Rather, hope that our results serve to stimulate discussion and future research aimed at further clarifying the mechanisms underlying letter spacing effects.

### Practical Implications of Letter Spacing Manipulations

The individual difference approach of our study expands previous findings, which suggested a dyslexia specific advantage of wider letter spacing (Zorzi et al., 2012; Bellocchi et al., 2019; Bertoni et al., 2019), to readers in a proficiency range that is generally considered normal (152–347 words per minute for standard letter spacing; see Brysbaert, 2019). Future studies should look at a broader range of reading proficiencies to specify under which conditions and for which readers the reduction of single word processing times (in terms of fixation durations) creates benefits that outweigh the disadvantages caused by increased numbers of fixations and reduced skipping. Given the widespread availability of digital text displays, the individually optimized adjustment of letter spacing is possible and could indeed provide quick and cost-efficient help for struggling readers. However, before widespread use of such tools is feasible, scientific understanding of who can benefit and under which conditions, has to be further substantiated. In the context of literacy acquisition, it has to be acknowledged as a potential risk that wider character spacing might allow for short term boosts in reading performance that may, however, prove disadvantageous in the long run because the reinforcement of slow serial letter processing can inhibit the development of a fast parallel recognition mode.

### Does Attention Mediate Crowding?

Lastly, although not in the focus of this study, it seems worth mentioning that we have observed a small advantage if crowded stimuli were presented in the periphery of the right visual field, even though participants fixated a central fixation point, as assured using eye-tracking. Even though there is general agreement that the anatomical locus of the crowding phenomenon lies in early visual processing areas (V1), there is growing evidence for additional top-down driven processes such as attention (Levi, 2008; Bertoni et al., 2019). Given that our sample of German readers normally reads from left to right, their visual span asymmetry expands to the right during reading (McConkie and Rayner, 1976) – which is in opposition to readers of right-to-left orthographies such as Hebrew (Pollatsek et al., 1981). This suggests that a reader’s dominant reading direction might influence habitual processes of attention allocation including parafoveal crowding sensitivity, which would be interesting to follow up in further research.

### Limitations

One aspect of our study that might be criticized is the relatively low number of participants (*N* = 24). Without any doubt, this study is to a substantial degree exploratory in nature, as an individual differences approach concerning the effect of letter spacing on reading rates (i.e., words per minute) in naturalistic, multi-line text presentations has so far not been investigated. A *post hoc* power analysis using the R package pwr (Champely, 2018) indicates that with a sample size of *N* = 24 a large correlational effect – as observed here for the association between wpm values during normal spacing with individual spacing effects (i.e., *r* = −0.62; see above Section “Reading Experiment – Words Per Minute”) – can be detected with a likelihood of 0.92. On the other hand, many of the correlations in eye tracking parameters (i.e., between random effects intercepts and slopes) were substantially smaller, ranging between 0.2 and 0.35, which indicates small to moderate effect sizes. To replicate these results, future studies will need to work with larger sample sizes.

Unsolved remains the issue that questions intended to control for the influence of text characteristics indicated (marginally) significantly higher ratings of prior knowledge in the normal letter spacing condition. Attempts to include prior knowledge ratings as a covariate in the LMMs did not succeed, since the increase in model complexity led to model convergence problems. Nevertheless, although we cannot rule out that prior knowledge had an impact on single word processing, our analysis approach, that is, linear mixed-effects modeling with crossed random effects for participants and words, took item specific characteristics on a single word level into account, which makes it rather unlikely that the reported letter spacing effects were primarily driven by prior knowledge.

## Conclusion

In summary, in line with previous eye-tracking experiments on paragraph reading in English (Slattery et al., 2016) and Spanish (Perea et al., 2016) we observed in a sample of young German readers without a history of reading impairments that wider character spacing produced robust effects of shorter fixation durations that did, however, not translate into reading rate gains. On the contrary, reading rates were slowed down, an effect that was stronger for faster readers and appears attributable to increased numbers of fixations under conditions of wider letter spacing. Neither foveal nor parafoveal crowding test scores correlated with individual differences in the letter spacing effect (in various measures). Strong effects during first fixation durations make it plausible that benefits from letter spacing are primarily driven by low level, foveal processes, whereas disadvantages especially for faster readers can be attributed to a disruption of fast, parallel letter processing of longer words. Additional components of the disadvantages on reading times are likely the result of reduced parafoveal preview benefit, the necessity for additional return sweeps in physically prolonged texts, and disturbed saccade programming. Importantly, our interpretation that wider spacing reinforces slow serial letter encoding, does not inevitably assume a source in the visual domain as a mediating factor for individual differences in response to wider letter spacing. In search for concrete advice on how wider letter spacing can be applied to improve reading performance, we suggest further research that jointly considers reading proficiency and susceptibility for word length effects.

## Data Availability Statement

Data and R-scripts used for analyses and for figure generation presented in the following are available at the OSF data repository: https://osf.io/vgze6/?view_only=e4fab3e77ba54d8d8e45b5551a5a449f..

## Ethics Statement

The studies involving human participants were reviewed and approved by Ethics Committee of the Department of Psychology at Goethe University Frankfurt (# 2014-80K). The patients/participants provided their written informed consent to participate in this study.

## Author Contributions

SK conceptualized the study, prepared the experimental design and stimuli, analyzed the data, and wrote the manuscript. KG contributed to the study concept, organized participant recruitment, collected data, and contributed to data analyses and interpretation. CF contributed to data interpretation and the writing of the manuscript.

## Conflict of Interest

The authors declare that the research was conducted in the absence of any commercial or financial relationships that could be construed as a potential conflict of interest.

## Footnotes

1 The rather high proportion of excluded participants is most likely due to the multiline text presentation instead of the more commonly used single sentence presentation in a narrow field around the horizontal midline of a computer screen. However, we opted for this presentation scheme to create a more ecologically valid reading situation.

## Appendix 1

Estimates of statistical significance for fixed effects in (generalized) linear mixed-effect models are provided through test statistics (i.e., *t*- or *z*-values). The significance for the inclusion of a random effect can be tested by comparing the model fit of a simpler model with a model with an additional random effect parameter but identical fixed effect structure. Table A1 provides goodness of fit indices for all five eye movement parameters in at least three levels of model complexity. Beginning with a model that included the fixed effect spacing condition we assumed only random intercepts for participants and words. We added complexity by including also random slopes for participants in the next model. And finally, the last model comprised random intercepts and random slopes for each word. For all duration measures (i.e., first fixation duration, gaze duration, and total reading time) models based on log-transformed values were compared.

The goodness of fit indices Akaike Information Criterion (AIC) and Bayesian Information Criterion (BIC) allow a direct interpretation, that is, the model with the lowest value is preferred. The difference of the log likelihood for a less complex model (lower *df* in Table A1) with a model of higher complexity multiplied by 2 follows a χ^2^ distribution with the difference of degrees of freedom between the compared model representing the degrees of freedom for the χ^2^ statistics. The *p*-values in the right column of Table A1 show that the full model always provided the best model fit. The only exception here is the eye movement parameter fixation count for which the full model led to overfitting.

**TABLE A1:** Three goodness of fit parameters (AIC, BIC, and log likelihood) for (G)LMMs of increasing random effect complexity.

Parameter	*df*	AIC	BIC	Log likelihood	χ^2^	χ^2^ *df*	*p*
First fixation duration	5	28311	28354	−14151			
	7	28295	28354	−14140	20.79	2	<0.001
	9	28072	28148	−14027	227.10	2	<0.001
Gaze duration	5	44294	44337	−22142			
	7	44290	44350	−22138	7.81	2	0.020
	9	44102	44179	−22042	192.44	2	<0.001
Total fixation time	5	51253	51296	−25622			
	7	51252	51312	−25619	5.18	2	0.075
	9	51167	51244	−25575	88.76	2	<0.001
Regression	4	31874	31908	−15933			
	6	31870	31921	−15929	8.56	2	0.014
	8	31781	31850	−15883	92.25	2	<0.001
Skipping	4	63247	63282	−31620			
	6	63140	63193	−31564	110.89	2	<0.001
	8	63085	63156	−31535	59.16	2	<0.001
Fixation count	4	126858	126893	−63425			
	6	126851	126905	−63420	10.24	2	0.006

**AIC, Akaike Information Criterion; BIC, Bayesian Information Criterion*.*

## Appendix 2

**TABLE A2:** Recalculation of correlations of visual acuity, foveal and parafoveal crowding with individual levels of spacing effects for six eye movement parameters, *p*-values in parentheses.

	Visual acuity	Foveal crowding	Parafoveal crowding intercept	Parafoveal crowding slope
First fixation duration	0.22 (0.32)	0.16 (0.48)	−0.24(0.28)	0.26 (0.22)
Gaze duration	0.17 (0.43)	0.09 (0.68)	−0.28(0.20)	0.26 (0.24)
Total fixation time	0.14 (0.53)	0.08 (0.73)	−0.20(0.35)	0.02 (0.92)
Regressions	−0.22(0.33)	−0.35(0.10)	0.02 (0.92)	−0.09(0.67)
Skipping	−0.22(0.33)	0.08 (0.72)	−0.06(0.80)	0.08 (0.70)
Fixation count	−0.04(0.86)	−0.22(0.30)	0.02 (0.93)	−0.21(0.33)

## References

[B1] Baayen R. H. Davidson D. J. Bates D. M. (2008). Mixed-effects modeling with crossed random effects for subjects and items. *J. Mem. Lang.* 59 390–412. 10.1016/j.jml.2007.12.005

[B2] Bates D. (2006). *Lmer*, *p-Values and All That.* Available at: https://stat.ethz.ch/pipermail/r-help/2006-May/094765.html (accessed June 15, 2019).

[B3] Bates D. Mächler M. Bolker B. Walker S. (2015). Fitting linear mixed-effects models using lme4. *J. Stat. Softw.* 67 1–48. 10.18637/jss.v067.i01

[B4] Bellocchi S. Massendari D. Grainger J. (2019). Effects of inter-character spacing on saccade programming in beginning readers and dyslexics. *Child. Neuropsychol.* 25 482–506. 10.1080/09297049.2018.1504907 30102106

[B5] Bertoni S. Franceschini S. Ronconi L. Gori S. Facoetti A. (2019). Is excessive visual crowding causally linked to developmental dyslexia? *Neuropsychologia* 130 107–117. 10.1016/j.neuropsychologia.2019.04.018 31077708

[B6] Bouma H. (1970). Interaction effects in parafoveal letter recognition. *Nature* 226 177–178. 10.1038/228549a0 5437004

[B7] Brysbaert M. (2019). How many words do we read per minute? A review and meta-analysis of reading rate. *J. Mem. Lang.* 109:104047 10.31234/osf.io/xynwg

[B8] Brysbaert M. Vitu F. Schroyens W. (1996). The right visual field advantage and the optimal viewing position effect: on the relation between foveal and parafoveal word recognition. *Neuropsychology* 10 385–395. 10.1037/0894-4105.10.3.385

[B9] Champely S. (2018). *Basic Functions for Power Analysis.* Available at: https://cran.r-project.org/package=pwr (accessed June 15, 2019).

[B10] Cohen L. Dehaene S. Vinckier F. Jobert A. Montavont A. (2008). Reading normal and degraded words: contribution of the dorsal and ventral visual pathways. *Neuroimage* 40 353–366. 10.1016/j.neuroimage.2007.11.036 18182174

[B11] Colenbrander A. (2008). The historical evolution of visual acuity measurement. *Vis. Impair. Res.* 10 57–66. 10.1080/13882350802632401

[B12] Coltheart M. Rastle K. Perry C. Langdon R. Ziegler J. (2001). DRS: a dual route cascaded model of visual word recognition and reading aloud. *Psychol. Rev.* 108 204–256. 10.1037/0033-295X.108.1.204 11212628

[B13] Dotan S. Katzir T. (2018). Mind the gap: increased inter-letter spacing as a means of improving reading performance. *J. Exp. Child Psychol.* 174 13–28. 10.1016/j.jecp.2018.04.010 29883749

[B14] Frömer R. Dimigen O. Niefind F. Krause N. Kliegl R. Sommer W. (2015). Are individual differences in reading speed related to extrafoveal visual acuity and crowding? *PLoS One* 10:121986. 10.1371/journal.pone.0121986 25789812 PMC4366391

[B15] Gagl B. Hawelka S. Wimmer H. (2015). On sources of the word length effect in young readers. *Sci. Stud. Read.* 19 289–306. 10.1080/10888438.2015.1026969 19628214

[B16] Haase W. Hohmann A. (1982). Ein neuer Test (C-Test) zur quantitativen Prüfung der Trennschwierigkeiten (“crowding”) - Ergebnisse bei Amblyopie und Ametropie [A new test (C-test) for quantitative examination of crowding with test results in amblyopic and ametropic patients]. *Klin. Monbl. Augenheilkd.* 180 2010–2015. 10.1055/s-2008-1055051 7078011

[B17] Hawelka S. Gagl B. Wimmer H. (2010). A dual-route perspective on eye movements of dyslexic readers. *Cognition* 115 367–379. 10.1016/j.cognition.2009.11.004 20227686 PMC2976468

[B18] Kliegl R. Wei P. Dambacher M. Yan M. Zhou X. (2010). Experimental effects and individual differences in linear mixed models: estimating the relationship between spatial, object, and attraction effects in visual attention. *Front. Psychol.* 1:238. 10.3389/fpsyg.2010.00238 21833292 PMC3153842

[B19] Korinth S. P. Fiebach C. J. (2018). Improving silent reading performance through feedback on eye movements: a feasibility study. *Sci. Stud. Read.* 22 289–307. 10.1080/10888438.2018.1439036

[B20] Levi D. M. (2008). Crowding-an essential bottleneck for object recognition: a mini-review. *Vis. Res.* 48 635–654. 10.1016/j.visres.2007.12.009 18226828 PMC2268888

[B21] Levi D. M. Klein S. A. Hariharan S. (2002). Suppressive and facilitatory spatial interactions in foveal vision: foveal crowding is simple contrast masking. *J. Vis.* 2 140–166. 10.1167/2.2.2 12678589

[B22] Li S. Oliver-Mighten L. Li L. White S. J. Paterson K. B. Wang J. (2019). Adult age differences in effects of text spacing on eye movements during reading. *Front. Psychol.* 9:2700. 10.3389/fpsyg.2018.02700 30671009 PMC6331398

[B23] McConkie G. W. Rayner K. (1976). Asymmetry of the perceptual span in reading. *Bull. Psychon. Soc.* 8 365–368. 10.3758/BF03335168

[B24] Pelli D. G. (2008). Crowding: a cortical constraint on object recognition. *Curr. Opin. Neurobiol.* 18 445–451. 10.1016/j.conb.2008.09.008 18835355 PMC3624758

[B25] Pelli D. G. Waugh S. J. Martelli M. Crutch S. J. Primativo S. Yong K. X. (2016). A clinical test for visual crowding. *F1000Research* 5:81 10.12688/f1000research.7835.1

[B26] Perea M. Giner L. Marcet A. Gomez P. (2016). Does extra interletter spacing help text reading in skilled adult readers? *Span. J. Psychol.* 19 1–7. 10.1017/sjp.2016.28 27210581

[B27] Perea M. Gomez P. (2012a). Increasing interletter spacing facilitates encoding of words. *Psychon. Bull. Rev.* 19 332–338. 10.3758/s13423-011-0214-6 22351586

[B28] Perea M. Gomez P. (2012b). Subtle increases in interletter spacing facilitate the encoding of words during normal reading. *PLoS One* 7:47568. 10.1371/journal.pone.0047568 23082178 PMC3474730

[B29] Perea M. Moret-Tatay C. Gómez P. (2011). The effects of interletter spacing in visual-word recognition. *Acta Psychol.* 137 345–351. 10.1016/j.actpsy.2011.04.003 21545978

[B30] Perea M. Panadero V. Moret-Tatay C. Gómez P. (2012). The effects of inter-letter spacing in visual-word recognition: evidence with young normal readers and developmental dyslexics. *Learn. Instr.* 22 420–430. 10.1016/j.learninstruc.2012.04.001

[B31] Pollatsek A. Bolozky S. Well A. D. Rayner K. (1981). Asymmetries in the perceptual span for Israeli readers. *Brain Lang.* 14 174–180. 10.1016/0093-934X(81)90073-0 7272722

[B32] R Core Team (2018). *R: A Language and Environment for Statistical Computing.* Vienna: R Core Team.

[B33] Radach R. Kennedy A. (2013). Eye movements in reading : some theoretical context. *Q. J. Exp. Psychol.* 66 429–452. 10.1080/17470218.2012.750676 23289943

[B34] Rau A. K. Moeller K. Landerl K. (2014). The transition from sublexical to lexical processing in a consistent orthography: an eye-tracking study. *Sci. Stud. Read.* 18 224–233. 10.1080/10888438.2013.857673

[B35] Rayner K. (1998). Eye movements in reading and information processing: 20 years of research. *Psychol. Bull.* 124 372–422. 10.1037/0033-2909.124.3.372 9849112

[B36] Rayner K. Slattery T. J. Drieghe D. Liversedge S. P. (2011). Eye movements and word skipping during reading: effects of word length and predictability. *J. Exp. Psychol. Hum. Percept. Perform.* 37 514–528. 10.1037/a0020990 21463086 PMC3543826

[B37] Reichle E. D. Warren T. McConnell K. (2009). Using E-Z reader to model the effects of higher level language processing on eye movements during reading. *Psychon. Bull. Rev.* 16 1–21. 10.3758/PBR.16.1.1 19145006 PMC2629133

[B38] Schotter E. R. Angele B. Rayner K. (2012). Parafoveal processing in reading. *Attention, Perception, Psychophys.* 74 5–35. 10.3758/s13414-011-0219-2 22042596

[B39] Skottun B. C. Skoyles J. R. (2012). Interletter spacing and dyslexia. *Proc. Natl. Acad. Sci. U.S.A.* 109 E2958–E2958. 10.1073/pnas.1212877109 22961254 PMC3497831

[B40] Slattery T. J. Rayner K. (2013). Effects of intraword and interword spacing on eye movements during reading: exploring the optimal use of space in a line of text. *Attent. Percept. Psychophys.* 75 1275–1292. 10.3758/s13414-013-0463-8 23709061

[B41] Slattery T. J. Yates M. Angele B. (2016). Interword and interletter spacing effects during reading revisited: interactions with word and font characteristics. *J. Exp. Psychol. Appl.* 22 406–422. 10.1037/xap0000104 27936854

[B42] Song S. Levi D. M. Pelli D. G. (2014). A double dissociation of the acuity and crowding limits to letter identification, and the promise of improved visual screening. *J. Vis.* 14:3. 10.1167/14.5.3 24799622 PMC4021854

[B43] Spinelli D. De Luca M. Judica A. Zoccolotti P. (2002). Crowding effects on word identification in developmental dyslexia. *Cortex* 38 179–200. 10.1016/s0010-9452(08)70649-x 12056688

[B44] Tadin D. Nyquist J. B. Lusk K. E. Corn A. L. Lappin J. S. (2012). Peripheral vision of youths with low vision: motion perception, crowding, and visual search. *Investig. Ophthalmol. Vis. Sci.* 53 5860–5868. 10.1167/iovs.12-10350 22836766 PMC3428114

[B45] Tiffin-Richards S. P. Schroeder S. (2015). Word length and frequency effects on children’s eye movements during silent reading. *Vis. Res.* 113 33–43. 10.1016/j.visres.2015.05.008 26048684

[B46] Vinckier F. Naccache L. Papeix C. Forget J. Hahn-Barma V. Dehaene S. (2006). “What” and “where” in word reading: ventral coding of written words revealed by parietal atrophy. *J. Cogn. Neurosci.* 18 1998–2012. 10.1162/jocn.2006.18.12.1998 17129187

[B47] Vinckier F. Qiao E. Pallier C. Dehaene S. Cohen L. (2011). The impact of letter spacing on reading: a test of the bigram coding hypothesis. *J. Vis.* 11:8. 10.1167/11.6.8 21566152

[B48] Weiss B. Knakker B. Vidnyánszky Z. (2016). Visual processing during natural reading. *Sci. Rep.* 6 1–6. 10.1038/srep26902 27231193 PMC4882504

[B49] Zorzi M. Barbiero C. Facoetti A. Lonciari I. Carrozzi M. Montico M. (2012). Extra-large letter spacing improves reading in dyslexia. *Proc. Natl. Acad. Sci. U.S.A.* 109 11455–11459. 10.1073/pnas.1205566109 22665803 PMC3396504

